# EGCG antagonizes Bortezomib cytotoxicity in prostate cancer cells by an autophagic mechanism

**DOI:** 10.1038/srep15270

**Published:** 2015-10-16

**Authors:** Alice Modernelli, Valeria Naponelli, Maria Giovanna Troglio, Martina Bonacini, Ileana Ramazzina, Saverio Bettuzzi, Federica Rizzi

**Affiliations:** 1 Department of Biomedicine, Biotechnology and Translational Research, University of Parma, Via Volturno 39/a, 43125 Parma, Italy; 2 Centre for Molecular and Translational Oncology (COMT), University of Parma, Parco Area delle Scienze 11/a, 43124 Parma, Italy; 3 National Institute of Biostructure and Biosystems (INBB), Viale Medaglie d’Oro 305, 00136 Rome, Italy

## Abstract

The proteasome inhibitors Bortezomib (BZM) and MG132 trigger cancer cell death via induction of endoplasmic reticulum (ER) stress and unfolded protein response. Epigallocatechin gallate (EGCG), the most bioactive green tea polyphenol, is known to display strong anticancer properties as it inhibits proteasome activity and induces ER stress. We investigated whether combined delivery of a proteasome inhibitor with EGCG enhances prostate cancer cell death through increased induction of ER stress. Paradoxically, EGCG antagonized BZM cytotoxicity even when used at low concentrations. Conversely, the MG132 dose-response curve was unaffected by co-administration of EGCG. Moreover, apoptosis, proteasome inhibition and ER stress were inhibited in PC3 cells simultaneously treated with BZM and EGCG but not with a combination of MG132 and EGCG; EGCG enhanced autophagy induction in BZM-treated cells only. Autophagy inhibition restored cytotoxicity concomitantly with CHOP and p-eIF2α up-regulation in cells treated with BZM and EGCG. Overall, these findings demonstrate that EGCG antagonizes BZM toxicity by exacerbating the activation of autophagy, which in turn mitigates ER stress and reduces CHOP up-regulation, finally protecting PC3 cells from cell death.

Cellular homeostasis and intracellular signaling pathways depend on the activity of proteins that are involved in almost all the cell functions: signaling, movement, transport, membrane fusion or cell protection are only some of them. Many quality control mechanisms contribute to the maintenance of protein homeostasis (proteostasis), in order to minimize dangerous consequences caused by misfolded or unnecessary proteins, or those synthesized in excess^1^. The proteasome, which is part of the protein quality system, is a highly conserved and essential system for degrading the majority of intracellular proteins in the eukaryotic cell^2^. The proteasome degradates short-lived regulatory proteins that play important roles in cell cycle, cell development and differentiation, DNA repair, and tumorigenesis. The majority of these proteins is tagged by a covalently linked polyubiquitin chain by the ubiquitination system prior to being subjected to degradation by the proteasome. Due to the essential role of the proteasome in cell function, the inhibition of its activity has been of interest for the development of therapeutic agents for cancer treatment. Pharmacologic inhibition of proteasome induces cell death in several cultured malignant cell lines; remarkably the inhibition of this complex is preferentially toxic to tumor cells rather than to normal cells. Bortezomib (BZM), a peptide boronic acid derivative, is a selective, reversible inhibitor of the 26S proteasome complex, approved by the United States Food and Drug Administration for treatment of multiple myeloma and mantle cell lymphoma. BZM is currently being investigated as a potential therapeutic agent against other tumors including prostate adenocarcinoma (PCa)^3^,^4^. BZM induces growth arrest and apoptosis in both androgen-dependent and androgen-independent PCa cells; in addition, it suppresses tumor growth in PCa animal models^3^,^5^,^6^. MG132 (N-benzyloxycarbonyl-L-leucyl-L-leucyl-L-leucinal) is a peptide aldehyde-based molecule that binds covalently to the active site of the beta subunits of 20S proteasome, leading to effective inhibition of 26S proteasome complex activity^7^. MG132 inhibits the growth of human PCa cells in nude mice^8^,^9^. Cell death triggered by proteasome inhibitors is associated with induction of endoplasmic reticulum (ER) stress, activation of the unfolded protein response (UPR), inhibition of the nuclear factor kappa B (NF-kB) inflammatory pathway, activation of caspase-8 and apoptosis, and increased generation of reactive oxygen species (ROS)^10^,^11^. Recent studies indicate that cell death induced by BZM and MG132 is a consequence of the accumulation of unfolded/damaged proteins culminating in activation of the ER stress response (ESR)^12^,^13^. The ESR is involved in alleviating the stressful disturbance and restoring proper ER homeostasis; however, in the case of intense or persistent ER stress, this pathway triggers UPR and programmed cell death^14^.

EGCG, the most abundant and bioactive polyphenol in green tea, displays strong preventive effects against cardiovascular disease, metabolic syndrome, neurodegenerative diseases and cancer^15^. In fact green tea extracts and EGCG promote growth inhibition and cell death in various human cancer cell lines and inhibit tumor incidence in numerous animal models, including TRAMP mice^16^,^17^,^18^, a murine transgenic model of PCa. Previously, we provided evidence that a standardized preparation of green tea catechins effectively prevented PCa in a cohort of men at high risk to develop the malignancy^19^. Proteasome inhibition is one of the mechanisms underlying the anticancer properties of EGCG^20^,^21^,^22^. Moreover, green tea extracts dramatically reduce the glycosylation capacity of ER, impacting the post-translational mechanism of protein maturation *in vivo* and *in vitro*^23^. Polyphenon E^®^, a standardized green tea extract mainly composed of EGCG (65%), exerts its antitumor effect on PCa cells by inducing ER stress, which in turn activates UPR associated signals^24^. In addition, EGCG binds to the ATP binding domain of the chaperone molecule glucose-regulated protein 78 (GRP78) blocking its function and promoting the activation of the ESR^12^,^13^.

Consistently with the above considerations, a rational assumption is that the anticancer properties of proteasome inhibitors could be enhanced by co-treatment with EGCG. By contrast, green tea polyphenols antagonize the anticancer effect of BZM in multiple myeloma cells by a direct interaction with the molecule’s boronic moiety^25^,^26^. Nevertheless, other Authors reported that BZM potentiates EGCG-mediated myeloma cell growth inhibition and apoptosis induction^27^. Besides these contradicting findings in multiple myeloma cells, it is noteworthy to mention that no antagonistic interaction between BZM and EGCG occurred *in vivo*, when a preclinical PCa model was used^28^. In this study, we examined the effects of the combination of boronic and non-boronic proteasome inhibitors (i.e., BZM and MG132, respectively) and sub-lethal doses of EGCG on androgen-independent PCa cell line (PC3 cells). We specifically investigated the impact of these combinations on molecular mechanisms involved in the control of protein homeostasis, induction of stress response and commitment to cell death.

## Results

### EGCG differently affects BZM- and MG132-induced cytotoxicity in human PCa cells

**Figure 1:**
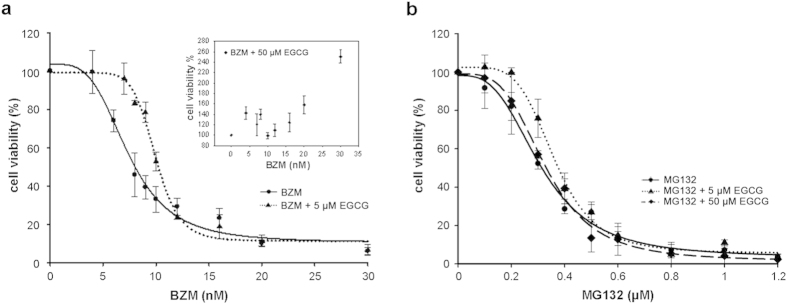
EGCG counteracts BZM- but not MG132-induced cytotoxic effects. Percentage of PC3 cell viability was determined by WST-1 assay after a 48 hour-treatment with increasing concentrations of BZM (**a**) or MG132 (**b**) in the presence or absence of EGCG (5 or 50 μM). Data are expressed as mean ± SD of four determinations in triplicate. Dose-response curves were generated and IC_50_ values were determined by non-linear regression analysis (four parameter logistic curve).

**Figure 2:**
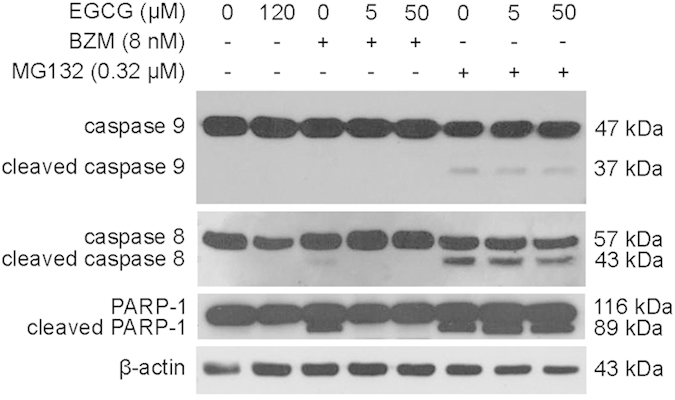
EGCG counteracts BZM- but not MG132-induced apoptosis. PC3 cells were treated for 48 hours with IC_50_ doses of EGCG, BZM or MG132. BZM and MG132 were tested in the presence or absence of EGCG (5 or 50 μM). Caspase-9, −8 and cleaved PARP-1 protein expression levels were analyzed by Western blot. β-actin was used as a loading control.

To evaluate whether BZM or MG132 cytotoxicity is affected by EGCG, PC3 cells were treated with increasing concentrations of each proteasome inhibitor in the presence or absence of EGCG (5 or 50 μM). Cell viability was determined after 48 hours of treatment with the WST-1 assay. Both BZM and MG132 reduced cell viability in a concentration-dependent manner with IC_50_ values of 8 nM and 0.32 μM, respectively (Fig. 1). The IC_50_ value of EGCG was 120 μM under similar experimental conditions (Supplementary Fig. 1). Co-administration of 5 μM EGCG resulted in a considerable shift to the right of the dose-response curve of BZM, whereas 50 μM EGCG completely suppressed BZM cytotoxicity (Fig. 1a). MG132 cytotoxicity was not substantially affected by EGCG, regardless of its concentration (Fig. 1b). The antagonistic effect exerted by EGCG on BZM-induced cell death was also evidenced by immunoblot analysis of apoptosis markers. A 48 hour-treatment with MG132 in the presence or absence of EGCG (5 or 50 μM) effectively activated cellular caspase-9 and −8, and induced cleavage of poly (ADP ribose) polymerase 1 (PARP-1) (Fig. 2). Conversely, BZM together with EGCG markedly reduced the cleavage of caspase-8 and PARP-1 relative to BZM as single agent. No cleaved caspase-9 was detected in BZM-treated PC3 cells regardless of whether EGCG was added. Moreover, no activation of caspase-9 and −8 or PARP-1 was shown in PC3 cells treated with IC_50_ doses of EGCG (Fig. 2). These results demonstrate that EGCG reduces BZM but not MG132 cytotoxicity in PC3 cells.

### EGCG prevents proteasome inhibition and ER stress induction in BZM-treated cells

**Figure 3:**
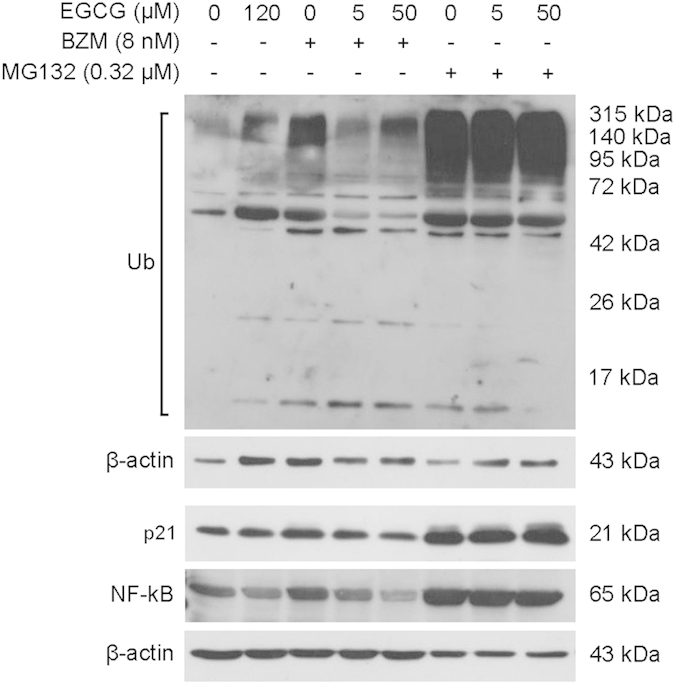
EGCG prevents proteasome inhibition when combined with BZM. PC3 cells were treated for 48 hours with IC_50_ doses of EGCG, BZM or MG132. BZM and MG132 were tested in the presence or absence of EGCG (5 or 50 μM). Western blot was used to detect polyubiquitinated proteins (Ub), p21 and NF-kB expression. β-actin was used as a loading control.

To determine the impact of EGCG on the capacity of BZM or MG132 to inhibit the proteasome, the level of polyubiquitinated proteins, p21 and NF-kB, which are proteins typically degraded via the ubiquitin-proteasome pathway, was verified by immunoblot analysis (Fig. 3). Our data confirmed the inhibition of the proteasome by both BZM and MG132, and to a lesser extent by EGCG (IC_50_ doses). Co-administration of 5 or 50 μM EGCG strongly prevented BZM- but not MG132-induced accumulation of polyubiquitinated proteins, p21 and NF-kB.

**Figure 4:**
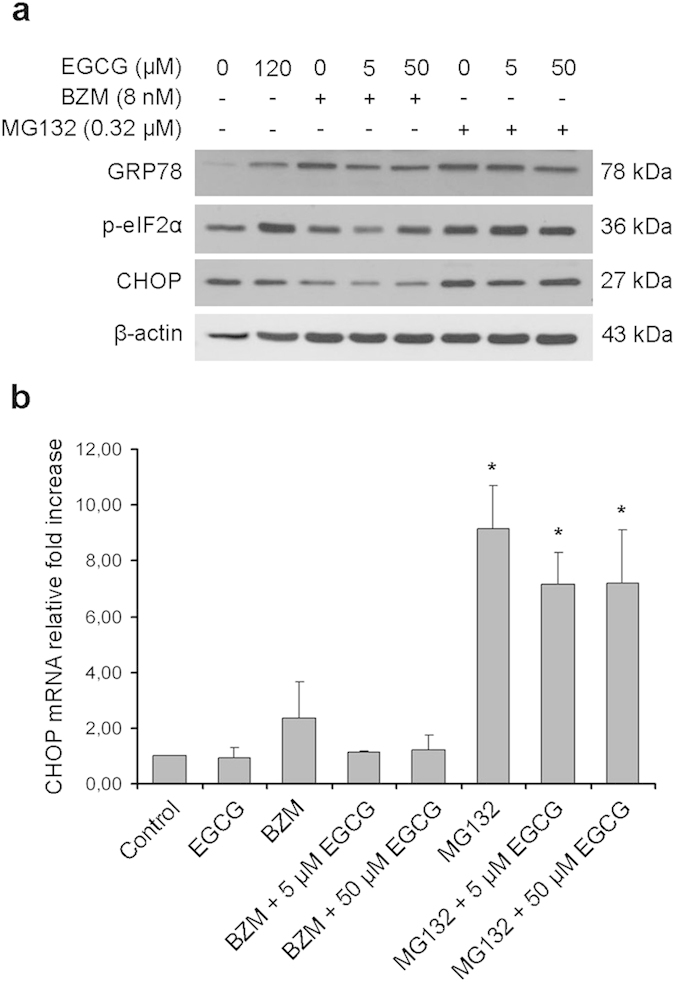
EGCG prevents ER stress induction when combined with BZM. PC3 cells were treated for 48 hours with IC_50_ doses of EGCG, BZM or MG132. BZM and MG132 were tested in the presence or absence of EGCG (5 or 50 μM). (**a**) Protein expression levels of the ER stress markers GRP78, CHOP and p-eIF2α were determined by Western blot, using β-actin as a loading control. (**b**) The expression levels of CHOP mRNA were determined by Real-Time quantitative reverse transcription PCR in the same samples. Results are expressed as mean ± SD of three determinations. One-way ANOVA test followed by Holm-Sidak multiple comparison test was used to compare treatment groups, *P < 0.001 relative to control.

Thereafter, the impact of the aforementioned treatments on triggering ER stress was analysed by detecting its well-documented molecular markers^29^, i.e. GRP78, CCAAT/enhancer binding protein homologous protein (CHOP) and the phosphorylated form of the initiation factor 2-alpha (p-eIF2α). GRP78, is a well known sensor of ER stress, CHOP is an ER-stress associated proapoptotic marker, and eIF2α is a translation initiation factor phosphorylated by protein kinase RNA-like endoplasmic reticulum kinase (PERK) in response to ER stress. The expression levels of GRP78 and CHOP increased in PC3 cells treated for 48 hours with MG132 (Fig. 4a). Combining BZM with EGCG (5 or 50 μM) resulted in a reduction of these ER stress markers relative to BZM alone. By contrast, EGCG did not affect ER stress induced by MG132, as demonstrated by consistently high levels of GRP78 and CHOP. Remarkably, p-eIF2α increased in PC3 cells treated with MG132 regardless of whether EGCG was added, whereas BZM did not considerably modify p-eIF2α levels and the co-administration of EGCG further reduced the phosphorylation of eIF2α. The protein expression data for CHOP was confirmed by Real-Time quantitative reverse transcription PCR (Fig. 4b). BZM treatment caused a slight increase in CHOP mRNA levels relative to controls (not significant), while the addition of EGCG (5 or 50 μM) resulted in a reduction of CHOP mRNA to basal levels. In PC3 cells treated with MG132 with or without EGCG (5 or 50 μM), CHOP mRNA levels increased significantly relative to control cells (P < 0.001), without substantial differences between cells treated with and without EGCG.

### EGCG and BZM combination promotes autophagy induction in PCa cells

**Figure 5:**
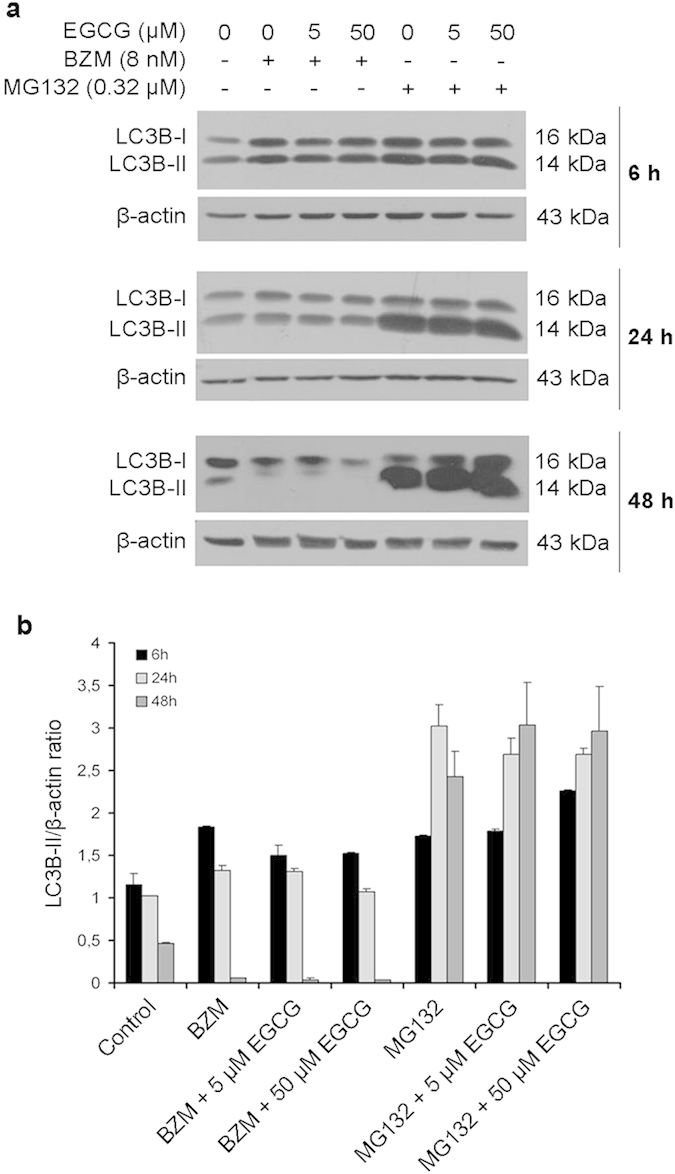
Proteasome inhibitors and EGCG promote LC3B-I to LC3B-II conversion. PC3 cells were treated for 6, 24 and 48 hours with IC_50_ doses of EGCG, BZM or MG132. BZM and MG132 were tested in the presence or absence of EGCG (5 or 50 μM). (**a**) Western blot analyses were performed to evaluate LC3B conversion (LC3B-I to LC3B-II). β-actin was used as a loading control. (**b**) Relative intensities of LC3B-II bands were quantified by densitometric analysis. Results are expressed as mean ± SD of three determinations.

**Figure 6:**
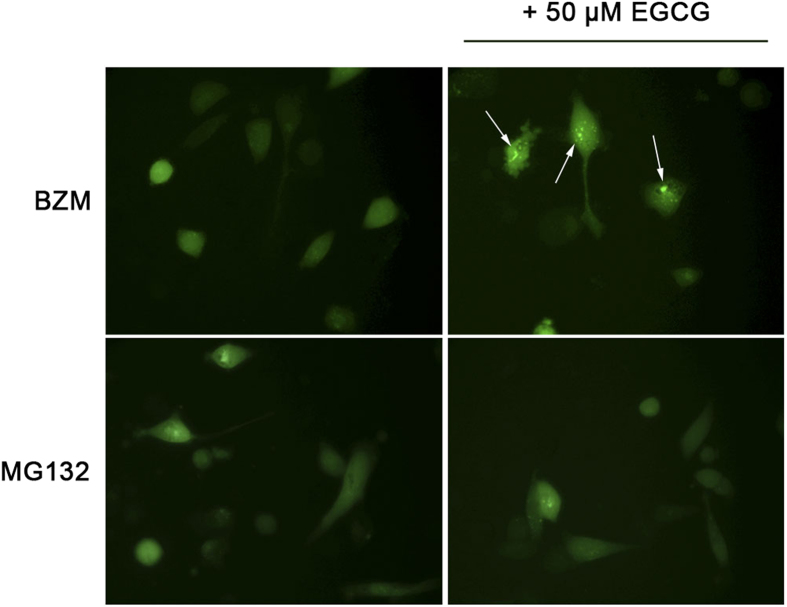
EGCG increases GFP-LC3 puncta formation in cells treated with BZM, but not with MG132. PC3 cells were transfected with GFP-LC3 and 20 hours after transfection were treated with IC_50_ doses of EGCG or IC_50_ doses of BZM or MG132 in the presence or absence of 5 or 50 μM EGCG. The appearance of GFP-LC3 puncta was observed for 48 hours using the Zeiss Axiovert 200 inverted fluorescence microscope. The images represent GFP-LC3 fluorescence observed after 6 hours of treatment with BZM or MG132, as single agents or combined with 50 μM EGCG (the arrows indicate GFP-LC3 puncta).

As autophagy can be activated following proteasome inhibition and is proposed as a mechanism of drug resistance, its role in cells subjected to proteasome inhibitors and EGCG was assessed. The microtubule-associated protein 1 light chain-3B (LC3B) is recruited from the cytosol and associates with the phagophore membrane early in autophagy. The conversion of LC3B-I (the cytosolic form) to LC3B-II (the lipidated form) correlates with the induction of autophagy and autophagosome formation^30^. As autophagy is a dynamic process immunoblot analyses were performed at 6, 24 and 48 hours after treatment. BZM and MG132 induced LC3B transition in PC3 cells already after 6 hours of treatment, regardless of whether EGCG was administered (Fig. 5a). In PC3 cells treated with MG132 with or without EGCG, LC3B-II accumulated up to 48 hours, whereas BZM in the presence or absence of EGCG substantially reduced LC3B-II levels over the same period, suggesting its degradation via lysosomal turnover (Fig. 5a,b). Another hallmark of autophagy is the redistribution of LC3 from a diffuse cytoplasmic pattern to a punctate cytoplasmic localization corresponding to autophagosomes. Therefore, we analysed the appearance of positive LC3 puncta in PC3 cells transiently transfected with GFP-LC3 plasmid DNA as a function of treatment period. PC3 cells treated with BZM or MG132 alone revealed diffuse cytoplasmic GFP-LC3 fluorescence staining, with a few visible puncta (Fig. 6). Punctuate expression of GFP-LC3 was dramatically increased after 6 hours of treatment with BMZ and 50 μM EGCG (Fig. 6); the same autophagic puncta were not visible in cells treated with MG132 and 50 μM EGCG.

**Figure 7:**
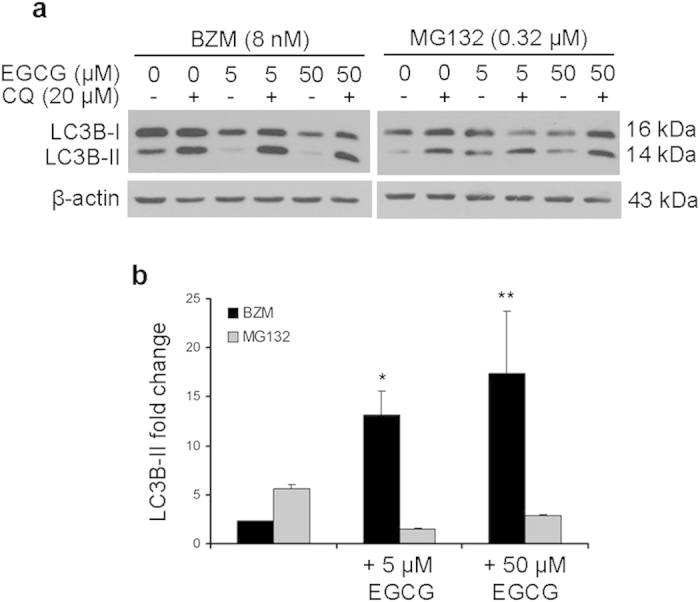
Chloroquine effects on LC3B-II accumulation. PC3 cells were treated for 24 hours as indicated. (**a**) LC3B-II accumulation was analyzed by Western blot, using β-actin as a loading control. (**b**) Relative intensities of LC3B-II bands (normalized against the level of β-actin) were quantified by densitometric analysis. LC3B-II accumulation in the presence of chloroquine (CQ) was expressed as fold change relative to LC3B-II levels in the absence of CQ. Results are expressed as mean ± SD of three determinations. One-way ANOVA test followed by Holm-Sidak multiple comparison test was used to compare treatment groups, **P < 0.001 and *P < 0.05 versus single treatment.

Both induction of autophagic flux and inhibition of autophagosomal degradation can cause an accumulation of LC3B-II or GFP-LC3 puncta^30^. Therefore, to correctly interpret our results, conversion of LC3B-I to LC3B-II was determined in the presence of chloroquine, a lysosomotropic agent. In the case of an efficient autophagic flux, chloroquine administration causes an enhancement of the accumulation of LC3B-II due to inhibition of autolysosome degradation^30^. Chloroquine co-administration increased LC3B-II transition elicited after a 24 hour-treatment with proteasome inhibitors, regardless of whether EGCG was co-administered (Fig. 7a,b). Remarkably, chloroquine induced considerably higher increase in LC3B-II levels in PC3 cells treated with BZM with or without EGCG relative to co-administration of MG132 and EGCG (Fig. 7a,b). Taken together, these results demonstrate that the co-administration of EGCG caused an enhancement of the autophagic flux activation above all when associated with BZM.

### Autophagy inhibition enhances BZM-induced cytotoxicity

**Figure 8:**
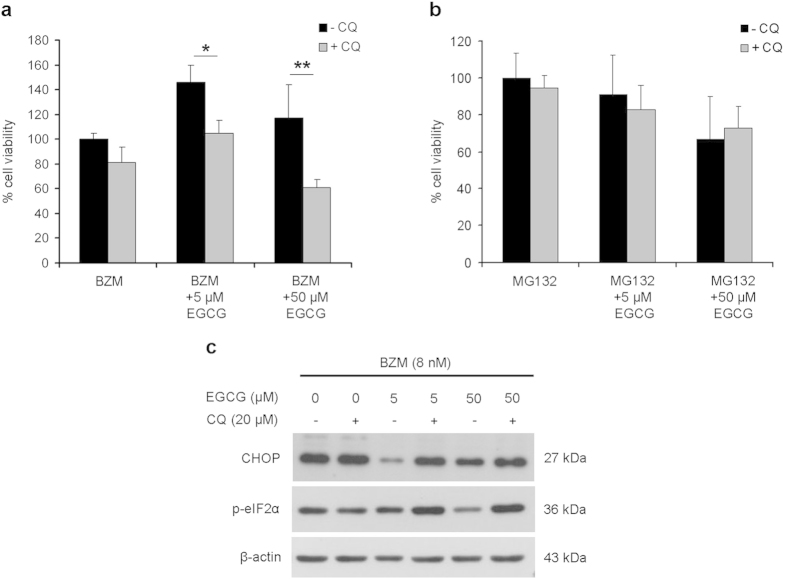
Autophagy inhibition rescues cytotoxicity and ER stress in PC3 cells treated with BZM and EGCG. PC3 cells were treated for 24 hours as indicated. Percentage of cell viability in the presence or absence of chloroquine (CQ) was determined by ATPlite assay. The viability of cells treated with BZM (**a**) or MG132 (**b**) as single agents was set as 100%. Results are expressed as mean ± SD of three determinations in triplicate. One-way ANOVA test followed by Holm-Sidak multiple comparison test was used to compare treatment groups, **P < 0.001 and *P < 0.05. (**c**) Western blot was performed to evaluate CHOP and p-eIF2α expression after 48 hours of the indicated treatments. β-actin was used as a loading control.

As autophagy can promote cell survival, the impact of autophagy on cytotoxicity induced by proteasome inhibitors in the presence or absence of EGCG (5 or 50 μM) was assessed by evaluating cell viability after co-administration of chloroquine. After 24 hours of combined treatment, cell viability was analysed by the ATPlite assay (Fig. 8a,b). Blocking autophagy with chloroquine significantly enhanced the cytotoxicity induced by the co-treatment with BZM and EGCG. Conversely, chloroquine did not affect the viability of PC3 cells treated with MG132, regardless of whether EGCG was co-administered. In addition, we evaluated the effect of autophagy inhibition on ER stress markers. In particular, the administration of 5 μM EGCG was more effective than the 50 μM dose on the suppression of CHOP. We observed a different effect on the expression of p-eIF2α, that was suppressed by EGCG in a dose dependent fashion. According to the cell viability data, chloroquine restored the BZM-mediated increase in CHOP and p-eIF2α protein levels that were reduced by administration of EGCG (Fig. 8c).

## Discussion

The critical contribution of the ESR to tumor cell growth and survival has only very recently begun to be recognized. The ESR follows a yin-yang principle, where low levels of cellular stress trigger its cell-protective module, whereas high levels of stress activate its pro-apoptotic machinery^31^,^32^. One of the central pro-survival regulators of the ESR is GRP78, which has important roles in protein folding and assembly, targeting misfolded protein for degradation, ER Ca^2 + ^binding and control of transmembrane ER stress sensor activation^33^. Conversely, CHOP is the most critical player of the pro-apoptotic arm of the ESR^34^. In case of intense or continuously high stress, pro-apoptotic proteins (such as CHOP and caspase-4) will gain dominance on the adaptive pro-survival functions and lead to cell death. Pharmacological strategies that combine two molecules with the aim to aggravate ER stress in cancer cells might imbalance the ratio between the pro-apoptotic and the pro-survival arm of the UPR in favor of cell death induction, possibly sensitizing apoptotic-resistant cells to alternative type of programmed cell death.

The UPR is associated with the antitumor activity of some flavonoids, including green tea catechins^35^. Polyphenon E^®^, a standardized green tea extract consisting mostly of EGCG, induces CHOP up-regulation in PC3 cells committing them to necroptosis, a type of programmed cell death not dependent on caspase activation^24^. Proteasome inhibitors, such as BZM or MG132, exert anticancer effects triggering ER stress in a wide variety of cancer cell lines, including PCa cells^36^. Notably, a phase II clinical trial revealed that BZM is not effective as a single agent for castration resistant metastatic PCa^37^. Therefore, we investigated the possibility that aggravation of ER stress via the simultaneous application of two different ESR-triggering drugs culminates in increased tumor cell death. Here, PC3 cells were treated with IC_50_ doses of BZM and MG132, two proteasome inhibitors representative of boronic- and non-boronic-based class compounds, as single agents or in combination with sub-lethal doses of EGCG. EGCG at concentrations of 5 and 50 μM was tested as the former represents concentration attained in human plasma^38^ and the latter corresponds to a non-apoptotic concentration for PC3 cells. This approach allowed for evaluation of EGCG on BZM- or MG132-induced cytotoxicity in an experimental model where approximately half of the cells have not already undertaken an irreversible cell death pathway.

When applied as single-drug treatments, BZM and MG132 reduced PC3 cells viability in a dose-dependent manner (IC_50_ values of 8 nM and 0.32 μM, respectively). We also confirmed that PC3 cells are relatively insensitive to EGCG treatment (IC_50_ = 120 μM), providing support that green tea polyphenols are chemopreventive rather than chemotherapeutic compounds, which target cells within an early ‘window of opportunity’ along the multistep progression toward advanced cancer^24^,^39^. Similarly, both BZM and MG132 triggered caspase and PARP cleavage committing cells to apoptotic death, while 120 μM EGCG had no effect. Remarkably, an EGCG concentration as low as 5 μM exerted nearly complete protection against BZM- but not MG132-induced cytotoxicity, findings in agreement with studies using human multiple myeloma cell lines^25^, suggesting that green tea consumption or EGCG supplementation should be avoided in BZM-treated patients.

To understand the molecular mechanisms underlying EGCG ability to counteract BZM but not MG132 toxicity in PC3 cells, the effect of the combined treatments on proteasome activity and ER stress induction were investigated. Proteasome inhibition was estimated by measuring the intracellular accumulation of both polyubiquitinated proteins and a short-lived protein known to be rapidly degraded by the proteasome, such as p21^40^. In addition, we monitored the levels of NF-kB, whose protein turnover is affected by both ubiquitination and proteasome-dependent degradation of the p65 subunit, events necessary for the efficient termination of the NF-kB transcriptional activity^41^,^42^,^43^. BZM and MG132, and to a lesser extent EGCG led to an accumulation of polyubiquitinated proteins, p21 and NF-kB relative to the control treatment. EGCG-mediated proteasome inhibition is well described^20^,^21^,^22^. Co-administration of EGCG and BZM reduced the accumulation of polyubiquitinated proteins as an indirect consequence of the relief of BZM-induced proteasome inhibition. The levels of p21 and NF-kB was also reduced under similar conditions. By contrast, EGCG did not antagonize MG132-mediated proteasome inhibition.

The ubiquitin proteasome system controls degradation of misfolded proteins at the ER, as well as the turnover of specific target proteins. Misfolded proteins are exported from the ER into the cytosol by ER-associated protein degradation and subsequently destroyed by the ubiquitin proteasome system^44^. The proteasome inhibition leads to the accumulation of polyubiquitinated and misfolded proteins in the ER and causes ER stress^45^,^46^. In our experimental model, ER stress induced by BZM and MG132 was evidenced by the increased levels of ER stress markers, GRP78 and CHOP. BZM induces a unique type of ER stress relative to other ER stress inducers, characterized by an absence of phosphorylation of eIF2α, which is a key cytoprotective component of the UPR that attenuates protein translation and therefore reduces the ER protein load^47^. Accordingly, in PC3 cells BZM did not induce the phosphorylation of eIF2α, whereas MG132 highly increased the levels of p-eIF2α compared to control. Remarkably, the co-administration of EGCG and BZM further reduced the levels of p-eIF2α. The lack of polyubiquitinated protein accumulation and increased CHOP levels following simultaneous treatments of PC3 cells with BZM and EGCG was correlated with a cytoprotective effect demonstrated by the inhibition of caspases and PARP cleavage, and an higher cell viability relative to the single drug treatment. EGCG directly binds to the boronic acid moiety of proteasome inhibitors, thus it antagonizes the effect of BZM, but not of MG132^25^,^26^,^48^.

Autophagy and proteasome, originally regarded as independent pathways, are mechanistically linked and complementary related protein degradation systems, which act together to ensure the intracellular proteostasis^49^. ER stress can activate autophagy in order to alleviate the burden of unfolded proteins and aggregates, thus reducing ER stress and avoiding apoptosis induction^49^. Several natural compounds, including polyphenols, strongly induce autophagy in PCa cells^50^. We have recently reported that autophagy acts as a pro-survival response to overcome the ER stress induced by green tea catechins in PNT1a immortalized prostate epithelial cells^24^. Here, a net autophagic flux was induced by BZM in PC3 cells and exacerbated by EGCG co-administration. These effects were not evident in PC3 cells treated with MG132 with or without EGCG. Autophagy inhibition by chloroquine yielded decreased cell viability in BZM treatments, especially when it was combined with EGCG. Conversely, inhibition of autophagy in PC3 cells treated with MG132 with or without EGCG did not affect cell viability. Autophagy inhibition overcomes the antagonistic effect of EGCG on BZM cytotoxicity by restoring ESR, yielding induction of eIF2α phosphorylation and CHOP up-regulation.

Taken together, EGCG antagonizes BZM but not MG132 by exacerbating the activation of a compensatory autophagic degradation of proteotoxic aggregates, which in turn mitigates ER stress and reduces CHOP up-regulation, culminating in protection of PC3 cells from apoptosis. ER stress induction and ubiquitinated protein accumulation is a novel and promising approach to cancer therapy, nonetheless the pro-survival contribution supplied by the activation of autophagy plays a relevant role in the on-set of therapy resistance, and should be taken in due account when boronic proteasome inhibitors are used.

## Materials and Methods

### Reagents

BZM and MG132 were purchased from Millennium Pharmaceuticals, Inc. (Cambridge, MA, USA) and Sigma-Aldrich (Saint-Louis, MO, USA), respectively. BZM and MG132 were prepared as 10 mM stock solutions in DMSO, stored at −80 °C and upon thawing diluted in fresh cell culture medium immediately before use. EGCG and chloroquine were purchased from Sigma-Aldrich and were dissolved in sterile cell culture medium at the required concentration immediately before use.

### Cell growth conditions

PC3 cells were purchased from the American Type Culture Collection (Manassas, VA, USA) and maintained in DMEM/Ham’s F12 medium, i.e. a mixture (1:1) of Dulbecco’s Modified Eagle’s Medium: Ham’s F12 (Gibco^®^, Life Technologies, Carlsbad, CA, USA) supplemented with 10% FBS, 2 mM L-glutamine, 100 U/mL penicillin and 100 μg/mL streptomycin (Lonza, Basel, CH). Cells were maintained at 37 °C in humidified atmosphere supplied with 5% CO_2_. Cell harvesting was performed by a Trypsin/EDTA treatment as per the manufacturer’s protocol (Sigma-Aldrich).

### Cell viability assay

PC3 cells were seeded at 9 × 10^3^ cells per well of a 96-well plate and allowed to attach overnight. Thereafter, cells were treated with 200 μL of DMEM/Ham’s F12 medium supplemented with increasing concentration of EGCG (0–350 μM), BZM (0–20 nM), MG132 (0–1.4 μM) or the aforementioned concentrations of BZM or MG132 in combination with 5 or 50 μM EGCG. After 48 hours of treatment, PC3 cell viability was determined with the WST-1 assay (Roche, Lewes, UK). For this, the spent medium was removed and replaced with fresh medium (100 μL/well) supplemented with 10% WST-1 reagent. This assay is based on the reduction of tetrazolium salt WST-1 to formazan by mitochondrial dehydrogenases in metabolically active cells. After a 1 hour incubation at 37 °C, formazan dye production was determined at an absorbance of 450 nm with the EnSpire^®^ multimode plate reader (PerkinElmer, Waltham, MA, USA). Dose-response curves were generated and IC_50_ (50% inhibiting concentration) values were determined by non-linear regression analysis (four parameter logistic curve) using SigmaPlot software (version 12.0). IC_50_ values were used in all subsequent experiments.

### ATPlite assay

PC3 cells were seeded in a 96-well microplate at a density of 1 × 10^4^ cells/well, and treated 24 hours later with 200 μL of medium supplemented with BZM or MG132 (for both inhibitors 0, 5 or 50 μM EGCG was co-administered) in the presence or absence of chloroquine. After 48 hours of treatment, the Luminescence ATP Detection Assay System (ATPlite) (PerkinElmer) was performed according to the manufacturer’s protocol. ATP monitoring can be used to assess the cytocidal, cytostatic and proliferative effects of drugs. Briefly, a 50 μL aliquot of mammalian cell lysis solution was added to each well and the plate was subsequently shaken at 700 rpm for 5 minutes in an orbital shaker. A volume of 50 μL/well of substrate solution was added and the microplate was shaken again for 5 minutes. After a 10 minute dark incubation period, the luminescence intensity was measured with EnSpire^®^ multimode plate reader (PerkinElmer).

### Protein extraction and Western blot analysis

PC3 cells (5 × 10^5^ cells/dish) were seeded in 60 mm dishes. After 48 hours, treated and untreated cells were washed twice with ice-cold PBS and scraped into ice-cold RIPA lysis buffer (50 mM Tris-HCl, pH 8.0, 1% Triton X-100, 150 mM NaCl) containing protease and phosphatase inhibitor cocktails (Sigma-Aldrich). Protein concentration was estimated by the DC Protein assay kit (Bio-Rad, Hercules, CA, USA), using bovine serum albumin (Sigma-Aldrich) as a standard. Total cell lysates (75 μg of protein in Laemmli buffer) were resolved by 12% acrylamide SDS-PAGE under reducing conditions and electrophoretically transferred to PVDF membranes (EMD Millipore, Merck KGaA, Darmstadt, DE). Transfer efficiency was routinely monitored by 0.1% Ponceau S (Sigma-Aldrich). Non-specific binding to the PVDF membrane was minimized by blocking for 1–5 hours at ambient temperature with TBS-T buffer (50 mM Tris-HCl, pH 7.5, 150 mM NaCl, 0.1% Tween 20) containing 5% (w/v) non-fat dry milk or 1% Blocking reagent solution (Roche, Basel, CH). PVDF membranes were incubated overnight with primary antibodies in 5% non-fat dry milk or 1% Blocking reagent solution at 4 °C with gentle shaking. Membranes were probed with the following primary antibodies: rabbit polyclonal anti-GRP78/BiP (GL-19) (dilution 1:100) and mouse monoclonal anti-p21^waf1/Cip1^ clone CP74 (dilution 1:200) from Sigma-Aldrich; rabbit polyclonal anti-ubiquitin (dilution 1:200), mouse monoclonal anti-NF-kB p65 (F-6) (dilution 1:200), mouse monoclonal anti-PARP-1 (F-2) (dilution 1:1,000) and mouse monoclonal anti-β-actin (dilution 1:500) from Santa-Cruz Biotechnology (Santa Cruz, CA, USA); mouse monoclonal anti-caspase 8 (1C12) (dilution 1:500), rabbit polyclonal anti-caspase 9 (dilution 1:1,000), mouse monoclonal anti-CHOP (L63F7) (dilution 1:1,000) and rabbit monoclonal anti-LC3B (D11) (dilution 1:1,000) from Cell Signaling Technology (Danvers, MA; USA); rabbit monoclonal anti-p-eIF2α (dilution 1:500) from Abcam (Cambridge, UK). Following the incubation with primary antibody, membranes were probed with horseradish peroxidase-conjugated anti-mouse (dilution 1:5,000) or anti-rabbit secondary antibodies (dilution 1:200,000) (Sigma-Aldrich) for 1 hour at ambient temperature. Immunoreactive bands were detected using the BM Chemiluminescence Western Blotting Substrate (POD) (Roche). Densitometric analysis of protein bands was performed using Quantity One^®^ 1-D Analysis software (version 4.6.3) (Bio-Rad).

### RNA extraction and Real-Time quantitative reverse transcription PCR

PC3 cells were seeded in 60 mm dishes at a density of 5 × 10^5^ cells/dish. After 48 hours of drug treatments, cells were harvested and dissolved in 1 mL TRIzol Reagent (Ambion^®^, Life Technologies). Total RNA was extracted according to the manufacturer’s protocol. Upon spectrophotometric quantification, 2 μg RNA aliquots were routinely electrophoresed on a 1% agarose gel to verify RNA quality and integrity. For reverse transcription reaction, ImProm-II™ Reverse Transcription System kit (Promega, Fitchburg, WI, USA) was used following the manufacturer’s protocol. Briefly, 1 μg of total RNA was combined with 1 μL of Oligo dT primers (0.5 μg/μL) and heated at 70 °C for 5 min. Thereafter, following a brief chill on ice, the Reverse Transcription mix was incubated at 25 °C for 5 min. First strand synthesis reaction was performed for 60 min at 42 °C. For each cDNA preparation, a 2 μL aliquot was used as template for Real-Time quantitative PCR. Primers sequences used were the following: CHOP/GADD153-fw: 5′-CTT CTC TGG CTT GGC TGA CT-3′, CHOP/GADD153-rv: 5′-TCC CTT GGT CTT CCT CCT CT-3′; GAPDH-fw: 5′-AAC CTG CCA AAT ATG ATG AC-3′,GAPDH-rv: 5′-TTG AAG TCA GAG GAG ACC AC-3′. PCR conditions were 40 cycles of 95 °C denaturing cycle (30 s), 63 °C annealing cycle (30 s) and a 72  °C extension cycle (30 s). Analysis of results was performed by DNA Engine Opticon 4 (MJ Research, Walthman, MA, USA) using the 2X SYBR Premix Ex Taq (Takara Bio Inc, Shiga, JP). PCR products were quantified by the relative quantification 2^-ΔΔCt^ method^51^ using GAPDH as a reference gene for data normalization. The relative abundance of transcripts in treated samples was expressed as fold change relative to controls.

### GFP-LC3 transient transfection

PC3 cells were seeded in 24-well plates at a density of 8 × 10^4^ cells/well and transfected with a GFP-tagged LC3 plasmid (GFP-LC3; Addgene plasmid # 11546)^52^ using FuGENE^®^ HD Transfection Reagent (Roche). Twenty hours after transfection, PC3 cells were treated as indicated and the formation of GFP-LC3 puncta in response to chemicals was observed for 48 hours thereafter using the Zeiss Axiovert 200 inverted fluorescence microscope equipped with the AxioCam HRc microscope color camera and the AxioVision 4.8 software (all from Carl Zeiss, Göttingen, DE).

### Statistical analysis

Data are expressed as mean values ± SD for the indicated number of independent determinations. All statistical analyses were performed using SigmaPlot software (version 12.0). One-way analysis of variance (One-way ANOVA) followed by a multiple comparison test was used to compare treatment groups; P values < 0.05 were considered statistically significant.

## Additional Information

**How to cite this article**: Modernelli, A. *et al*. EGCG antagonizes Bortezomib cytotoxicity in prostate cancer cells by an autophagic mechanism. *Sci. Rep*. **5**, 15270; doi: 10.1038/srep15270 (2015).

## Supplementary Material

Supplementary Information
