# Supplementary material for: EGCG antagonizes Bortezomib cytotoxicity in prostate cancer cells by an autophagic mechanism
Source: Sci Rep. 2015 Oct 16;5:15270. doi: 10.1038/srep15270 (PMC4607952; doi:10.1038/srep15270)
Supplement: Supplementary Information [file srep15270-s1.pdf]

EGCG antagonizes Bortezomib cytotoxicity in prostate cancer cells by an autophagic mechanism

Alice Modernelli[1,2#], Valeria Naponelli[1-3#], Maria Giovanna Troglio[1], Martina Bonacini[1], Ileana Ramazzina[1-3], Saverio Bettuzzi[1-3*] and Federica Rizzi[1-3*].

[1]Department of Biomedicine, Biotechnology and Translational Research, University of Parma, Via Volturno 39/a, 43125 Parma, Italy; [2]Centre for Molecular and Translational Oncology (COMT), University of Parma, Parco Area delle Scienze 11/a, 43124 Parma, Italy; [3]National Institute of Biostructure and Biosystems (INBB), Viale Medaglie d'Oro 305, 00136 Rome, Italy.

[#] These authors contributed equally to the article.

* Corresponding authors: Federica Rizzi, e-mail: federica.rizzi@unipr.it; Saverio Bettuzzi, e-mail: saverio.bettuzzi@unipr.it.

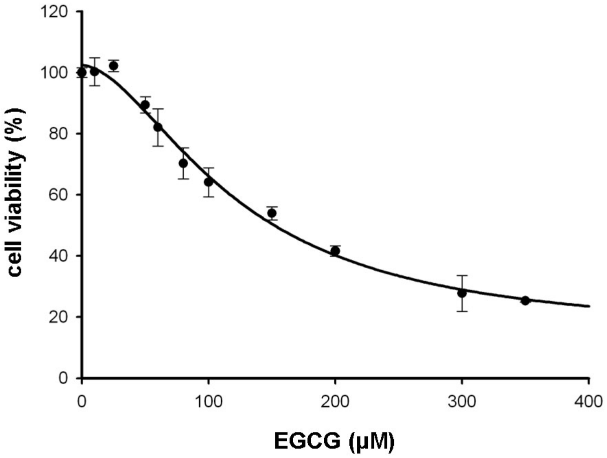

Supplementary Figure 1 – Dose-response curve of EGCG. PC3 cells were treated 48 hours with increasing concentrations of EGCG. Percentage of PC3 cell viability was determined by WST-1 assay. Data are expressed as mean ± SD of three determinations in triplicate. The dose-response curve was generated and IC_{50} value was determined by non-linear regression analysis.
